# Use of Tunable Whole-Cell Bioreporters to Assess Bioavailable Cadmium and Remediation Performance in Soils

**DOI:** 10.1371/journal.pone.0154506

**Published:** 2016-05-12

**Authors:** Youngdae Yoon, Sunghoon Kim, Yooeun Chae, Yerin Kang, Youngshim Lee, Seung-Woo Jeong, Youn-Joo An

**Affiliations:** 1 Department of Environmental Health Science, Konkuk University, Seoul 05029, Korea; 2 Division of Bioscience and Biotechnology, BMIC, Konkuk University, Seoul 05029, Korea; 3 Department of Environmental Engineering, Kunsan National University, Kunsan 54150, Korea; East China Normal University, CHINA

## Abstract

It is important to have tools to measure the bioavailability to assess the risks of pollutants because the bioavailability is defined as the portions of pollutants showing the biological effects on living organisms. This study described the construction of tunable *Escherichia coli* whole-cell bioreporter (WCB) using the promoter region of zinc-inducible operon and its application on contaminated soils. It was verified that this WCB system showed specific and sensitive responses to cadmium rather than zinc in the experimental conditions. It was inferred that Cd(II) associates stronger with ZntR, a regulatory protein of zinc-inducible operon, than other metal ions. Moreover, the expression of reporter genes, *egfp* and *mcherry*, were proportional to the concentration of cadmium, thereby being a quantitative sensor to monitor bioavailable cadmium. The capability to determine bioavailable cadmium was verified with Cd(II) amended LUFA soils, and then the applicability on environmental systems was investigated with field soils collected from smelter area in Korea before and after soil-washing. The total amount of cadmium was decreased after soil washing, while the bioavailability was increased. Consequently, it would be valuable to have tools to assess bioavailability and the effectiveness of soil remediation should be evaluated in the aspect of bioavailability as well as removal efficiency.

## Introduction

Environmental pollution caused by anthropogenic activities is considered a major threat to human health. Pollutants such as heavy metals that enter into the ecosystem can transfer to living organisms by diverse exposure routes, and pollutant accumulation has been shown to cause toxicity in humans with prolonged direct and indirect exposure [1–3]. Thus, efficient tools for monitoring and quantifying pollutants in the environment are needed. Traditionally, pollutants have been monitored by analytical instruments that are expensive and time-consuming to use. Moreover, these instruments are unable to distinguish bioavailable portions from unavailable pollutants in order to assess the risks of pollutants to living organisms.

As a response to these needs, whole-cell bioreporters (WCBs) were developed as alternative tools. WCBs are genetically engineered bacterial cells that respond to specific pollutants, such as toxic chemicals, antibiotics, and heavy metals [4–7]. In general, WCBs are constructed by the fusion of sensing domains, including the promoter region of a specific pollutant-responsive operon that regulates the expression of reporter genes, which indicates the exposure of bacterial strains to specific pollutants [5, 8]. In the case of heavy metals, genes from metal-responsive operons have been widely used for metal-sensing domains, and diverse proteins, including luminescent proteins [9], fluorescent proteins [10, 11], and enzymes such as β-galactosidase [12] able to show their expression directly, have been employed for reporter domains. Since the genetic characterization of specific metal-responsive operons and their regulatory mechanisms are readily available [13, 14], numerous bioreporters based on bacterial cells have been developed to detect heavy metals, including arsenic, mercury, copper, lead, chromium, and cadmium [10, 15–20]. Most of these bioreporters exhibit sub-nanomolar to micromolar detection ranges for heavy metals. In spite of the diverse metal-specific WCBs available, only a few have been applied to environmental samples [20–22]. This could be because of the lack of selectivity of bioreporters for heavy metals and the difficulty of sample preparation due to the complexity of environments. Thus, it is necessary to improve the heavy metal selectivity of bioreporters and to optimize experimental conditions in order to overcome the complexities of environmental samples for truly quantitative measurements using WCB assays.

The WCBs investigated here were constructed by the genetic fusion of the promoter/operator regions (zntAp) of the *znt*-operon (zinc-responsive operon) in *Escherichia coli* DH5α and reporter genes, enhanced green fluorescent protein (eGFP) and mCherry. Therefore, the transcription of reporter genes was regulated by endogenous ZntA and ZntR, which are regulatory proteins controlling the export of Zn(II), Cd(II), and Pb(II) ions from *E*. *coli* cells [23, 24]. The correlation between sensing elements and reporter genes is a critical feature of genuine WCB. We describe here the characterization of *E*. *coli* WCBs based on zntAp and report the bioavailability of cadmium in contaminated soils. WCBs showed cadmium-specific responses in the experimental conditions and it was revealed that substituting the reporter gene for another is a simple method for tuning the dynamic range of cadmium detection of WCBs. The cadmium bioavailability in soil samples was further investigated using the WCB assay.

## Materials and Methods

### Bacterial strain and materials

*E*. *coli* DH5α was used as the host strain for plasmid construction and as the recipient for the plasmids pZnt-eGFP and pZnt-mCherry. Heavy metal salts, including As_2_O_3_, Na_2_HAsO_4_, CdCl_2_, K_2_Cr_2_O_7_, CuCl_2_·2H_2_O, HgCl_2_, NiCl_2_, PbCl_2_, and ZnCl_2_, were purchased from Sigma-Aldrich (St. Louis, MO, USA) and used to prepare 10 mg/mL metal(loid) stock solutions. Landwirtschaftliche Untersuchungs und Forschungsanstalt (LUFA) standard soil (LUFA Speyer, Germany) was used for the preparation of cadmium-amended soil samples. Contaminated field soils before and after soil-washing were obtained from a smelter area in Korea (geographic coordinate of the site, 36.009300, 126.669701).

### Plasmid construction

**Fig 1:**
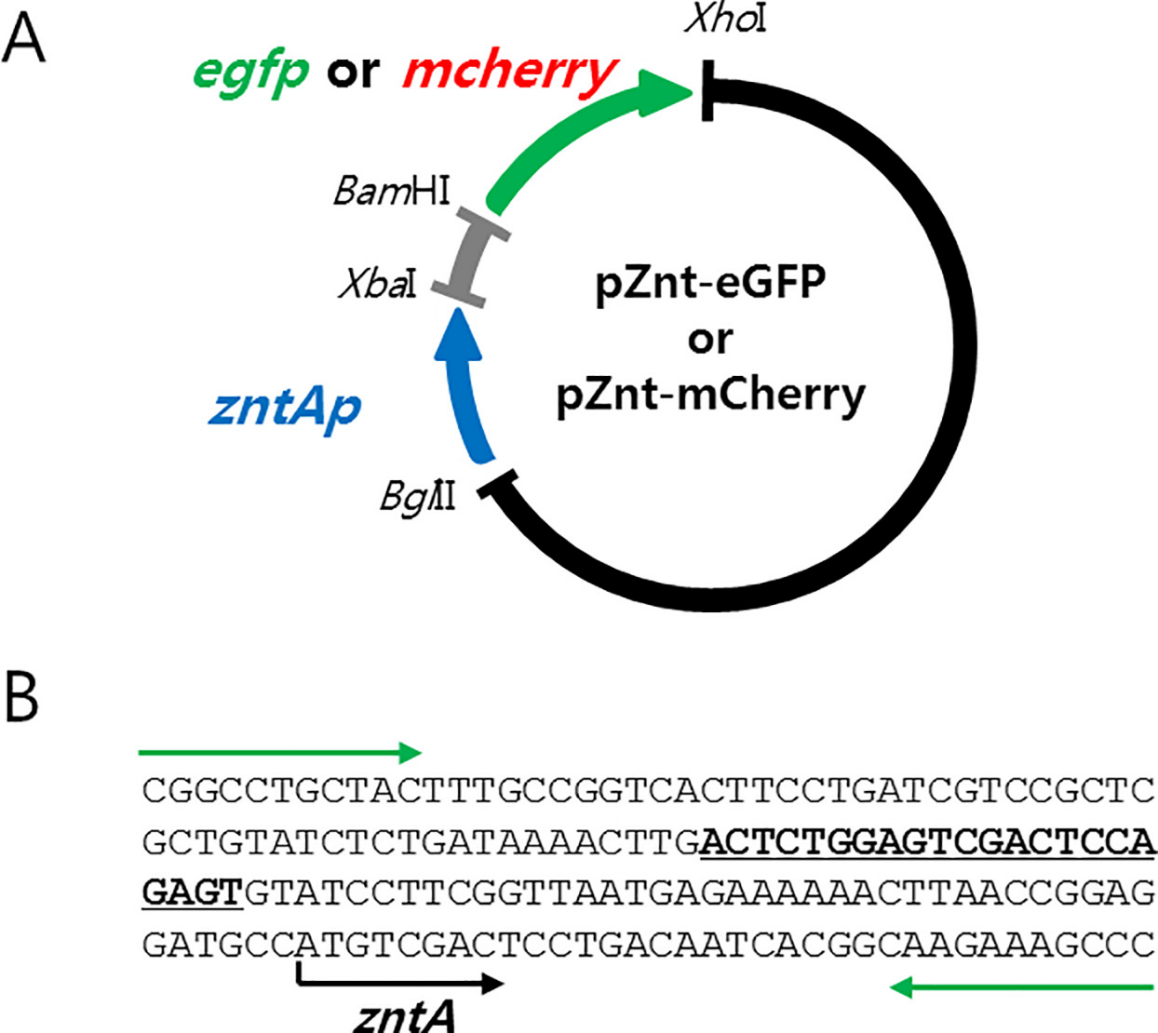
A schematic diagram of plasmid construction for WCBs for cadmium quantification. (A) The zntAp region amplified by PCR was inserted into pET21(a) with *Bgl*II and *Xba*I to replace the T7 promoter, and then *egfp* or *mcherry* was inserted downstream of the zntAp with *Bam*HI/*Xho*I to construct pZnt-eGFP and pZnt-mCherry. (B) The DNA sequence of zntAp region used for WCB plasmid construction. The ZntR binding site is shown as underlined bold letters. Green arrows indicate the primer pair used for PCR.

The promoter region of the *znt*-operon in *E*. *coli* (zntAp) was amplified by PCR from the genomic DNA of *E*. *coli* DH5α extracted by traditional alkaline lysis methods supplemented with lysozyme [25]. The sequences of the zntAp region and primers are shown in Fig 1. The amplified zntAp region was digested with *Bgl*II and *Xba*I restriction enzymes and inserted into pET-21(a) to replace the original T7 promoter region. Then, the genes encoding green and red fluorescent proteins amplified from pEGFP-N1 and pmCherry-1 (Clontech Laboratories, Inc.), respectively, were inserted downstream of zntAp with *Bam*HI and *Xho*I to generate pZnt-eGFP and pZnt-mCherry (Fig 1A). Then the plasmids were introduced into *E*. *coli* DH5α to generate WCBs.

### WCB assay

WCBs were grown overnight at 37°C in Luria–Bertani (LB) broth containing ampicillin (50 μg/mL), and then cells from the overnight culture were added to 50 mL of fresh LB broth. When the optical density value at 600 nm (OD_600_) reached 0.4, different concentrations of heavy metal ions were added. WCBs (1 mL) exposed to heavy metals were collected at different incubation times, and the cells were harvested by centrifugation. The cell pellets were resuspended in 1 mL of 50 mM Tris-HCl (pH 7.4) containing 160 mM KCl before the measurement to avoid interferences caused by the LB broth. The expression of fluorescent reporter proteins was determined using an FS-2 fluorescence spectrometer (Scinco, Korea). For the WCB assay, the bandwidth for excitation and emission was set to 5 nm, and the excitation/emission wavelengths were set to 470/510 nm and 575/610 nm for eGFP and mCherry, respectively. The induction of reporter protein was represented by the induction coefficient, defined as [fluorescent intensity of bioreporter with heavy metal]/[fluorescent intensity of bioreporter without heavy metal].

### Characterization of the WCBs

#### The relationship between cell growth and induction of reporter proteins

WCBs harboring pZnt-eGFP and pZnt-mCherry were grown at 37°C in the shaking incubator, and 5 mg/L of cadmium was added after 3 h. The OD_600_ values and the emission intensities of reporter proteins were monitored to investigate the relationship between cell growth and the induction of reporter proteins. A WCB cell sample was collected at different time frame during 12 hours from the beginning of the WCB assay to measure the cell density and induction coefficients using a UV spectrometer and fluorescence spectrometer, respectively.

#### Heavy metal selectivity test

The stock solutions of heavy metals were prepared by dissolving metal compounds in demineralized and sterilized water. The selectivity was determined by comparing the induction coefficient of reporter proteins induced by different heavy metals. WCBs harboring pZnt-eGFP were exposed to 5, 10 and 20 mg/L of heavy metals, and the induction coefficients at 1 and 3 h exposure were compared.

#### Determination of detection ranges

From the metal selectivity test, WCBs showed a specific response to cadmium, but not zinc, even though the sensing element, zntAp, originated from a zinc-inducible operon. To verify the superior sensitivity of WCB toward cadmium, tests were performed with both cadmium and zinc. The WCBs were exposed to different concentrations of cadmium and zinc, and the induction coefficients were determined for different exposure durations; Cadmium and zinc ranging from 0−5 mg/L and 0−30 mg/L were tested for WCBs harboring pZnt-eGFP and pZnt-mCherry, respectively. The induction coefficients at different exposure times were measured to compare sensitivities and to determine detection ranges for quantifying bioavailable cadmium.

### Analysis of bioavailable cadmium in soils

#### Sample preparation

Cadmium bioavailability in cadmium-amended LUFA soils and contaminated field soils was assessed using WCBs. The amended LUFA soils were prepared using the following protocol. Briefly, LUFA soils were spiked with several concentrations of cadmium at final concentrations of 0, 10, 20, 30, 40, and 50 mg/kg, and then samples were stored for more than 7 days. Contaminated field soils were collected from three sites from smelter areas in Korea before and after an acid-soil washing process and the physicochemical properties of site soils were characterized by standard protocols [26, 27]. We got permission from Korea Environment Corporation through Korean Ministry of the Environment to collect field samples for each site. A soil solution for each sample was prepared using previously reported protocols with minor modifications [28, 29]. Four bed volumes of sterilized water was added to soil samples and then mixed by shaking for 24 hours at room temperature. The mixtures were spun down to remove soil, and then the supernatants were filtered through Whatman filter paper twice. The soil solution was finally obtained after filtration through a nitrocellulose membrane (0.45 μm). The total amount of cadmium in all soil samples was determined via inductively coupled plasma-atomic emission spectroscopy (ICP-AES; JY 138; Ultrace, Jobin Yvon, France).

#### Quantification of bioavailable cadmium in soil

To determine bioavailable cadmium in soils, WCBs were exposed to soil directly. The soil (0.25 g) was added to 5 ml of WCBs with 0.4 of OD_600_ and then the induction coefficients were measured after 1 and 3 h of exposure. The induction coefficients were determined using a fluorescence spectrometer and *in vivo* and *in vitro* assays for whole cells and soluble fraction of WCB cells, respectively. The soluble fraction was obtained by lysing the cells by sonication, followed by centrifugation at 15,000 rpm to clarify the lysed cells. Briefly, the *in vivo* assay was used to measure the intensity of reporter proteins in whole *E*. *coli* cells, and the *in vitro* assay was used to measure the intensity of reporter proteins in the soluble fraction to eliminate interference from soil particles.

The standard curve for the *in vivo* assay was obtained by the following procedure: 5 ml of pre-incubated WCBs were added to test tubes containing 0.25 g of cadmium-free soil to correct for interferences caused by small particles of soil, and then cadmium solution was added to test tubes at a final concentration of 0 to 0.5 mg/L. After 1 and 3 h, WCBs (1 mL) were collected by 1 min of centrifugation at 1,500 × g to remove the majority of soil particles. The supernatants were centrifuged for 5 min at 15,000 × g to pellet WCB cells, which were then resuspended in 1 ml of Tris-HCl buffer (160 mM KCl and 50 mM Tris-HCl, pH 7.3) before measuring the fluorescence. The induction coefficient of each test unit after 1 and 3 h of exposure was plotted as a function of cadmium concentration. In the case of the *in vitro* assay, the soluble fraction was obtained by cell lysis via sonication in 1 ml of Tris-HCl buffer followed by centrifugation at 15,000 × g for 10 min. The induction coefficient of the soluble fraction of each test unit was plotted against the concentration to generate a standard curve. The amount of bioavailable cadmium in soil samples was calculated from the standard curves and converted to μg per 1 g of soil. Consequently, the values from the WCB assay was multiplied 20 times to obtain the amount of cadmium per 1 g of soil because 1/20 portion was analyzed during the quantification processes.

#### Quantification of bioavailable cadmium in soil solution

The *in vivo* assay was employed for quantification of bioavailable cadmium in soil solution because soil particles, which are major interfering factors, were excluded during the preparation. For the WCB assay, 2 ml of soil solution, which corresponded to 0.5 g of soil, was added to the test unit containing 3 ml of pre-incubated WCBs. The induction coefficient was measured after 1 and 3 h of exposure, and the bioavailable cadmium was determined based on a standard curve. The standard curve for the soil solutions was obtained by spiking known concentrations of cadmium ranging from 0−1 mg/L to WCB culture. The amount of bioavailable cadmium was converted to the amount (μg) per 1 g of soil for convenient comparison; thereby the values determined by WCB assay was multiplied 10 times because 1/10 portion of 1 g soil was analyzed.

### Structural analysis

The 3-dimensional crystal structure of ZntR, which regulates the znt-operon, was obtained from the Protein Data Bank (PDB ID: 1Q08). The ZntR structure is a homo-dimer, each monomer containing two zinc ions. The total energy of ZntR with different metal ions was calculated after energy minimization using the Tripos force field. The energy minimization process was terminated at the convergence criteria for the total energy (0.05 kcal/mol per Å). The total energies and 3-dimensional structures of ZntR bearing different metal ions were analyzed. The metal-binding site of ZntR was further analyzed to determine the number of protein-metal ion interactions and the distance between metal ions and ZntR using Ligplot (EMBL-EBI).

## Results and Discussion

### Metal selectivity of WCBs

**Fig 2:**
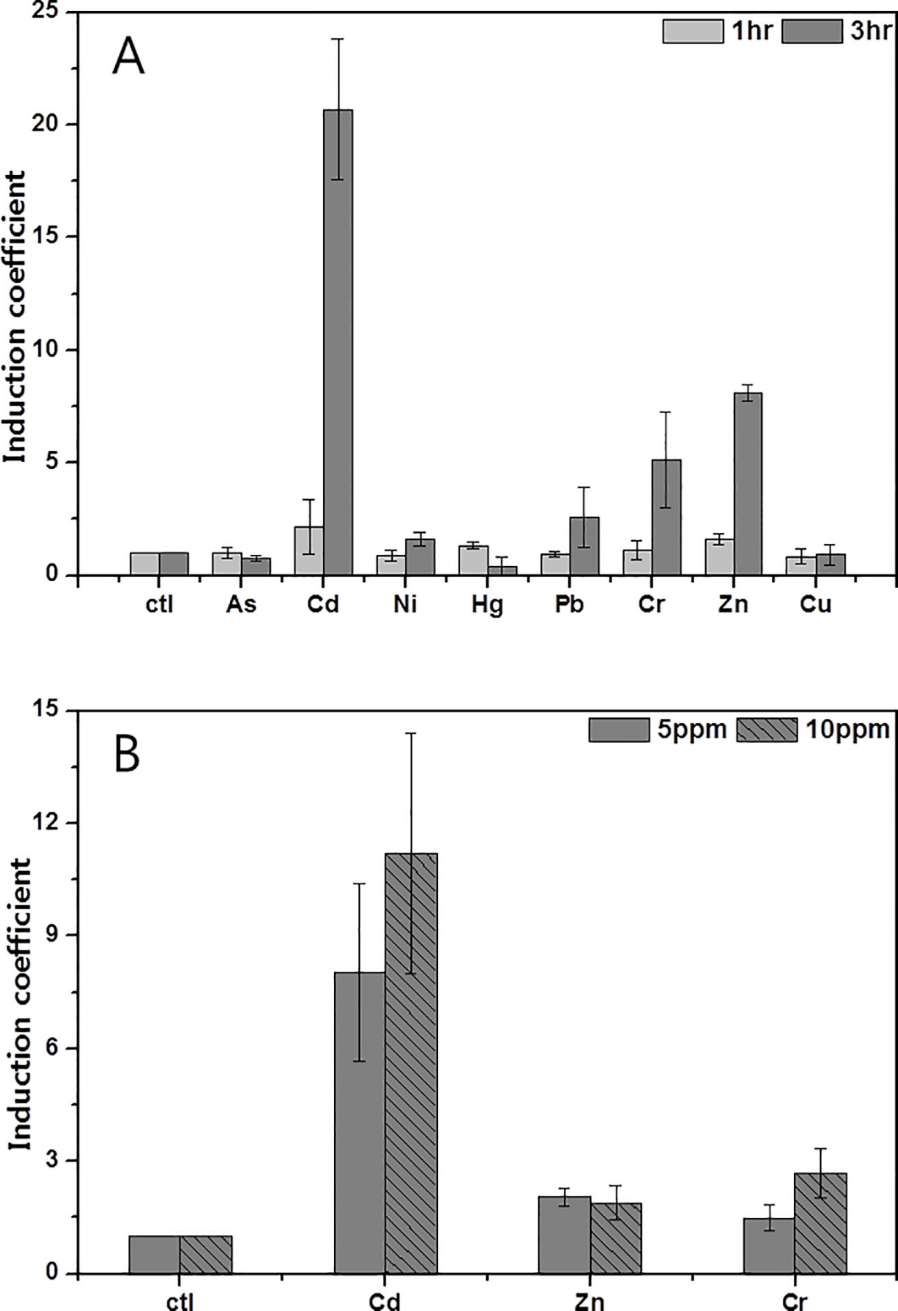
Heavy metal selectivity of WCBs harboring pZnt-eGFP. (A) The induction coefficient for eGFP was determined after 1 h (grey bar) and 3 h exposures (black bar) to 20 mg/L of 8 different metals and metalloid ions: As(III), Cd(II), Ni(II), Hg(II), Pb(II), Cr(VI), Zn(II), and Cu(II). (B)The induction coefficient of WCBs exposed to 5 mg/L (dark grey) and 10 mg/L (marked) Cd(II), Zn(II), and Cr(VI) for 3 h. The data are the mean values of three replicates with the standard deviations.

The metal selectivity of WCBs based on zntAp was investigated with As(III), Zn(II), Co(II), Cr(VI), Hg(II), Pb(II), Cu(II), and Cd(II). Both WCBs harboring pZnt-eGFP and pZnt-mCherry were tested and they showed same pattern of responses toward heavy metal ions. As shown in Fig 2, the WCB harboring pZnt-eGFP showed highest induction coefficient toward Cd(II) among 20 mg/L of tested metal(loid)s. However, the induction coefficient from Cd(II) was not dominant to others with 1 h exposure at 20 mg/L. It would be reason by 1 h exposure was not sufficient to form the mature eGFP. When the exposure duration was increased to 3 h, the induction coefficient from Cd(II) was increased from 2.1±1.24 to 22±3.12, and those of other metals such as Zn(II), Cr(VI) and Pb(II) were 8.09±0.36, 5.11±2.14 and 2.56±1.32, respectively (Fig 2A). To verify the capability to quantify bioavailable cadmium, the lower concentrations of Cd(II), Zn(II) and Cr(VI) were tested. Zn(II) and Cr(VI) showed 2.05±0.36 and 1.49±0.35 of induction coefficient values at 10 mg/L and 1.89±0.46 and 2.68±0.65 at 5 mg/L, respectively, while Cd(II) showed 11.2±3.21 and 8.03±2.35, respectively (Fig 2B). Although Zn(II) showed 4.2 and 5.4 times less responses than Cd(II) at 5mg/L and 10mg/L, respectively, it was necessary to confirm the effect of Zn(II) on WCBs because zntAp was known to respond to Zn(II). Moreover, zinc is known to present generally more abundant than cadmium in soils and the mobility and potential bioavailability are also higher [30]. Therefore, we included Zn(II) along with Cd(II) in the further experiments to rule out the effect of Zn(II)

Previously published cadmium-monitoring WCBs have employed *zntA* or *cadA1* genes from *E*. *coli* or *P*. *putida*, respectively, as sensing elements [9, 20, 31]. Despite their cadmium-sensing ability, they were not used as quantitative cadmium bioreporters because of their broad selectivity toward other metal ions. However, the response to cadmium was 200–500 times higher than zinc from the WCBs employing zntAp as sensing domain. It was inferred that the WCBs investigated here could be applied to cadmium quantification at the restricted conditions. As described above, the WCBs showed specific response to Cd(II) at low concentration with 1 h exposure duration. Therefore, we can quantify the bioavailable cadmium using the WCBs by adjusting the experimental conditions that was not sufficient for zinc to affect WCBs.

### Molecular analysis of heavy metal binding to ZntR

The structural analysis of the interaction between ZntR and metal ions such as Zn(II), Cu(II), Cr(VI) were carried out to have better understanding about metal selectivity of WCBs based on zntAp. The crystal structure of ZntR (PDB ID: 1Q08) consists of a homo-dimer, with each monomer containing two zinc ions (S1A Fig). One zinc ion (Zn1) was stabilized by His119, Cys115, phosphate, and Cys79 of the opposite monomer of ZntR; the other zinc (Zn2) was secured by Cys114, Cys124, phosphate, and Cys79 of the opposite monomer (S1B Fig). Since both monomeric chains were involved in stabilization of the metal ions, minimization was carried out as a dimer with four metal ions. When zinc, copper, or chromium ions were bound to ZntR, the total energy of the protein after minimization was -438.041, -438.480, or -435.255 kcal/mol, respectively. Then the distance between the residues in the metal binding site of ZntR and each metal ion was calculated and listed in S1 Table. Since the structural information was not available, the energy minimization was not performed with Cd(II). However, it was speculated that the Cd(II) would associate with ZntR more tighter than others because it has larger atomic and covalent radii; thereby the Cd(II)-ZntR complex would be more stable. It is understandable why the WCB employing zntAp was more sensitive to cadmium than the other metals tested. These results are further supported by recent reports on the substitution of divalent metal ions, such as Zn(II), Ni(II), Co(II), and Cd(II), in metal binding proteins [32–34]. In consequence, the quantification of bioavailable cadmium using WCBs described in the present study makes sense given that the interaction of the endogenous ZntR in *E*. *coli* with Cd(II) is predicted to be stronger than any other metal ions, including zinc.

### Cadmium toxicity test

To verify the toxic effects of cadmium on *E*. *coli*, the relationship between WCB cell density (optical density at 600nm) and expression of reporter genes in order to reliably use this WCB to quantify bioavailable cadmium in contaminated soils, The growth curves of WCBs treated by with and without 5 mg/L of cadmium were monitored, and there was no adverse effect was observed until 12 h (S2B Fig). Thus, toxic effects of cadmium on *E*. *coli* would be excluded from the WCB assay for the quantification of bioavailable cadmium. In addition, it was observed that eGFP was induced faster than mCherry by Cd(II) exposure (S2B Fig). The different response rates of both reporter proteins are probably caused by difference of maturation time to the active forms [35].

### Cadmium Sensitivity of WCBs

**Fig 3:**
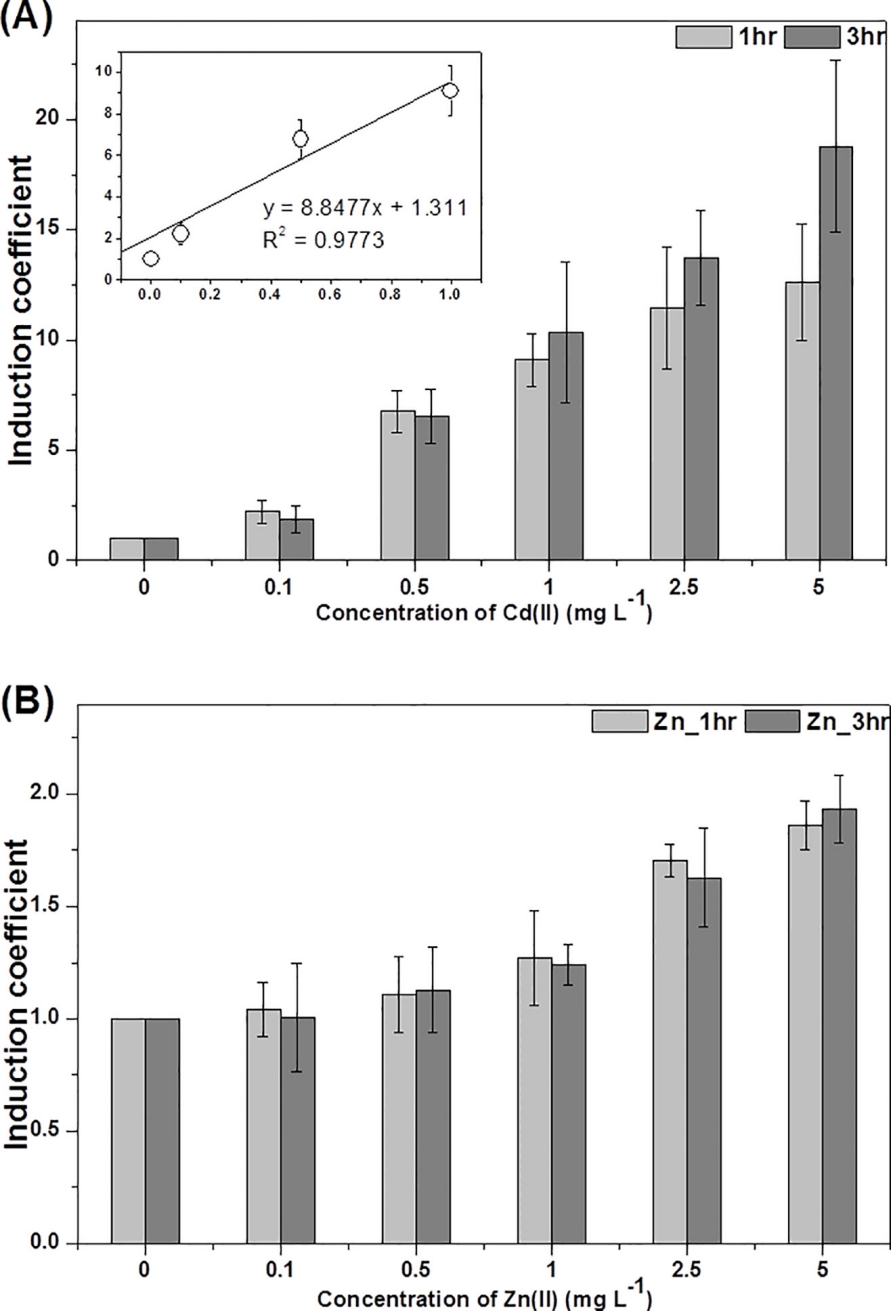
The responses WCBs harboring pZnt-eGFP exposed to different concentrations of Cd(II) and Zn(II) ions during 1 h (grey) and 3 h (dark grey) exposures. (A) The induction coefficients of WCBs increased as a function of Cd(II) ranging from 0–5 mg/L and it was fit well by linear regression with R^2^ = 0.9773 for Cd(II) ranging from 0–1 mg/L from 1 h exposure (inset). (B) The induction coefficients for WCBs exposed to Zn(II) ranging from 0–5 mg/L. The data are the mean values of three replicates with the standard deviations.

Most importantly, the sensitivity of WCBs, which determines their dynamic ranges, is a critical property for quantifying bioavailable heavy metals in environmental systems. Since the WCBs were based on the promoter region of a zinc-inducible operon, both Zn(II) and Cd(II) were tested. The WCBs were exposed to both Zn(II) and Cd(II) at final concentrations of 0, 0.1, 0.5, 1, 2.5, and 5 mg/L, and the induction coefficients were determined after 1 and 3 h of exposure. In concordance with previous results, the induction coefficients of Zn(II) ranged from 1–2, whereas the induction coefficients of Cd(II) ranged from 1–20 (Fig 3). It was noticed that the induction coefficient was proportional to the concentration of cadmium and fit to a linear regression curve with an R^2^ value of 0.9773 in 0–1 mg/L range of Cd(II) (Fig 3A inset). Thus, the bioavailable cadmium in contaminated soils was determined from the linear regression curve.

### Tuning dynamic ranges of WCBs by replacing reporter genes

**Fig 4:**
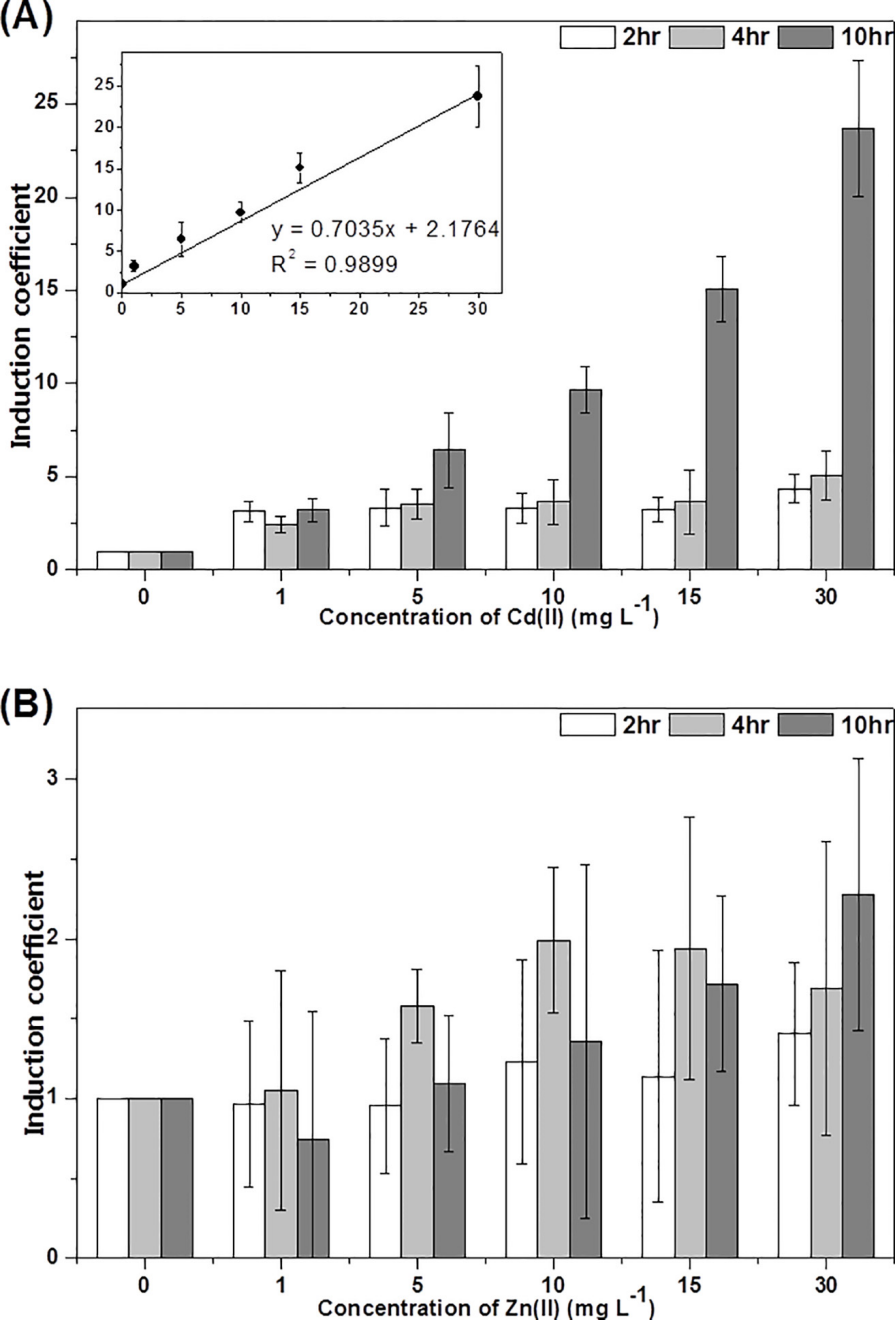
Induction coefficients for WCBs harboring pZnt-mCherry exposed to different concentrations of cadmium (A) and zinc (B), ranging from 0–30 mg/L for 2 h (white), 4 h (grey), and 10 h (dark grey) exposures. The inset shows the induction coefficients from 10 h exposure and Cd(II) concentrations ranging from 0–30 mg/L fit by linear regression with R^2^ = 0.9899. The data are the mean values of three replicates with the standard deviations.

To obtain a diversity of WCBs, two fluorescent proteins, *egfp* and *mcherry*, were employed as reporter genes. Although they are structurally similar proteins, the maturation time of eGFP is about 5 times faster than that of mCherry [35]. In concordance with this, pZnt-mCherry WCB produced slower and weaker response than pZnt-eGFP, even with higher concentrations and longer exposure of Cd(II) (Fig 4A). Although the induction coefficients from 2 and 4 h of exposure with 1 mg/L of Cd(II) were observed as 3.15±0.57 and 2.46±0.45, respectively, the values from short time exposure would not be used for the quantification because the Cd(II) concentration and induction coefficients were not correlated proportionally. On the other hand, it was fitted well to a linear regression from 10 h exposure (Fig 4A inset). Although this slow responding property of pZnt-mCherry WCBs would not be ideal for rapid and efficient detection of cadmium, it was notable to tune the dynamic range by replacing reporter genes. In addition, it was also verified that replacing reporter genes could not produce any effects on metal selectivity (Fig 4B).

Since the available WCBs are relatively limited, there have been many efforts to engineer existing WCBs to modulate properties, such as sensitivity and selectivity. The metal homeostasis system of host bacteria has been mutated to interfere with the efflux of metals to accumulate inside of cells, improving detection limits [17]. Another report showed that the reporter protein signal could be tuned by changing feedback circuitry including promoter and regulatory genes [20]. In addition to previous reports, we propose a new method for tuning the detection ranges of WCBs by replacing reporter genes. Since substitution of *egfp* with *mcherry* increased the dynamic range, it would be possible to tune the detection limit by introducing reporter genes having different maturation times.

### Quantification of bioavailable cadmium in soil samples

**Fig 5:**
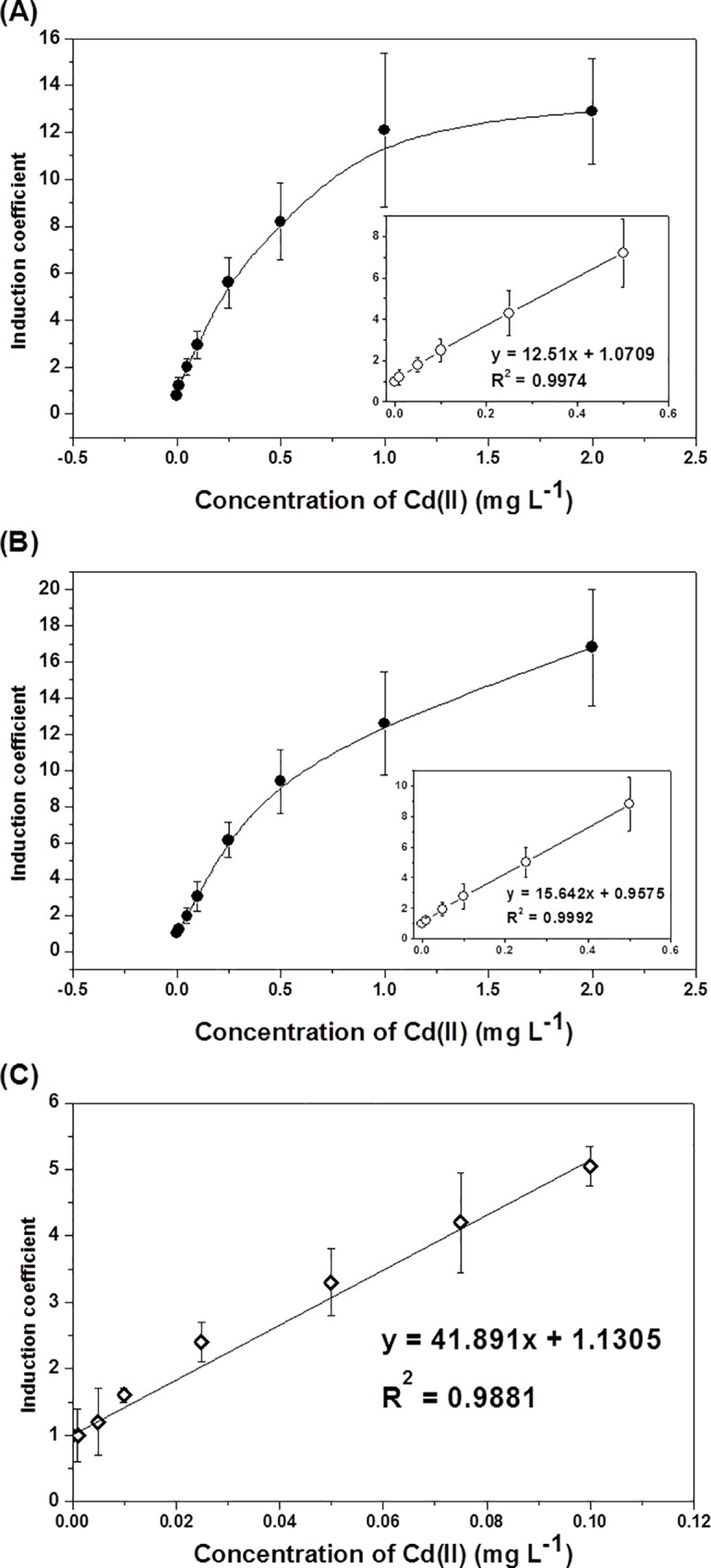
Standard curves for quantifying bioavailable cadmium in contaminated soils using the *in vivo* assay for soils (A), *in vitro* assay for soils (B), and *in vivo* assay for soil solutions (C). The insets in (A) and (B) show the equation for the linear regression for quantification of Cd(II) ranging from 0–0.5 mg/L. The data are the mean values of more than three replicates with the standard deviations.

The bioavailable cadmium in contaminated soils was determined using pZnt-eGFP WCB because it is more sensitive than pZnt-mCherry WCB. The contaminated soil samples were prepared as soils and soil solutions representing total and water-soluble cadmium, respectively. In the case of soil samples, the bioavailable cadmium was determined by two different WCB assays, the *in vivo* and *in vitro* assays, and only *in vivo* assay was used for soil solution samples. The amount of bioavailable cadmium in soil samples was calculated from the induction coefficients of WCBs based on the linear regression curves for data corresponding to Cd(II) concentrations ranging from 0–0.5 mg/L (Fig 5), and the quantification was carried out based on linear regression curves from induction coefficient of 1 h exposure.

#### Analysis of cadmium bioavailability in amended soils

The metal contents in LUFA soils was first determined to exclude the possible effects of metals present in LUFA soils. The heavy metal(loid)s including cadmium, arsenic, zinc, lead, nickel and copper, were analysed by ICP-AES, and only zinc and copper were detected as 0.254 and 0.058 mg/kg, respectively. Thus, it was supposed that the cadmium detected by WCB in amended LUFA soil samples was from the originally spiked Cd(II). The bioavailable and total cadmium in contaminated LUFA soil samples was determined using the WCB assay and ICP-AES, respectively, and is summarized in Table 1. The amount of bioavailable cadmium in amended LUFA soils was 8.42–17.56 mg/kg and 3.81–12.89 mg/kg, as measured using the *in vivo* and *in vitro* assays, respectively, and was 35–84% and 25–38% of the original amount of cadmium used to spike the samples. In this case, the cadmium bioavailability determined by the *in vitro* assay was about 45–73% less than by the *in vivo* assay. Nonetheless, it was hard to know which assay is more relevant to the actual amount of bioavailable cadmium because of a lack of standard methods to determine bioavailable cadmium. However, when adequate reference soils are not available, the *in vitro* WCB assay using soluble fractions is an alternative that excludes soil interference.

**Table 1:** Amount of total and bioavailable cadmium in amended LUFA soil samples*.

	Soil					Soil solution				
	total^†^	bioavailable cadmium				total		bioavailable cadmium		
		*in vivo*	% of total	*in vitro*	% of total	ICP-AES	% of total	*in vivo*	% of soluble total	% of total
	0	N.D.	-	N.D.	-	<LOD^‡^	-	N.D.	-	-
	10	8.42 ±0.99	84.2	3.81 ±0.65	38.1	0.22 ±0.02	2.23	0.015 ±0.035	29.69	0.66
**Amended**	20	12.18 ±0.75	60.9	7.92 ±1.06	39.6	0.24 ±0.04	2.44	0.034 ±0.067	38.36	0.59
**LUFA soil**	30	12.87 ±2.54	42.9	9.87 ±0.82	32.9	0.30 ±0.08	2.97	0.083 ±0.013	47.84	0.47
	40	14.92 ±2.76	37.3	12.05 ±1.65	30.1	0.39 ±0.07	3.87	0.201 ±0.019	67.84	0.65
	50	17.56 ±2.63	35.1	12.89 ±2.36	25.7	0.43 ±0.09	4.28	0.278 ±0.112	76.02	0.65

* The values were converted to μg of cadmium/g of soil and represented as average with standard deviation as errors from more than 3 replicated tests, N.D.; not determined, % of total was the percentage of determined amount out of total cadmium in samples.

^†^ The concentration of cadmium originally spiked in LUFA standard soil.

^‡^ The concentration was lower than instrumental limit of detection.

As listed in Table 1, the amounts of total and bioavailable cadmium in soil solutions were considerably lower than those in soils. The amount of total cadmium detected in soil solutions was only 2–4% of the original cadmium used to spike the samples, and bioavailable cadmium was 0.47–0.66%. This result suggested that the majority of bioavailable cadmium in soils was present as non-extractable forms strongly associated with soil particles. It was supported by the evidences that heavy metals are adsorbed to soil particles and are toxic to microorganisms [36, 37]. On the other hand, the fraction of bioavailable cadmium out of the total soluble cadmium was 30–76%. It was partially matched to general notion that water soluble cadmium is bioavailable. However, it was also noticed that certain portion of water soluble cadmium was not bioavailable.

#### Evaluation of remediation effectiveness by assessing cadmium bioavailability in contaminated field soils

The total and bioavailable amount of cadmium in contaminated field soils before and after soil-washing were investigated using the same methods described above, and detailed in Table 2. The induction coefficients of WCBs exposed to field soil samples and the amount of bioavailable cadmium was calculated from standard curves and converted to mg/kg (S3 Fig). The total amount of cadmium in soils and soil solutions decreased after soil-washing processes, but the bioavailable cadmium did not. At Site 1 in particular, the total cadmium in both the soil and soil solution decreased after soil washing, but bioavailable cadmium decreased in the soil and increased in the soil solution. However, the fraction of bioavailable cadmium increased by about 60% and 25% of the total in soil and soil solution, respectively. Hence, it was speculated that the soil-washing process removed some cadmium and released cadmium tightly associated with soils, making more cadmium bioavailable. At Site 2, the bioavailable cadmium increased in soil and decreased in soil solution after soil-washing. In other words, the absolute amount of bioavailable cadmium decreased after soil-washing, but the bioavailable fraction inconsistently changed at each site. The increase of bioavailable fraction after soil washing could be explained by that harsh conditions of soil-washing processes disrupted the association of cadmium with soils; thereby non-bioactive cadmium was transformed to bioactive Cd(II). Therefore, it was concluded that soil washing is effective for removing total cadmium, but not very effective for reducing bioavailability.

**Table 2:** Amount of total and bioavailable cadmium in contaminated field soils before and after soil-washing *.

		Soil					Soil solution				
		total	bioavailable				total		bioavailable		
		ICP-AES	*in vivo*	% of total	*in vitro*	% of total	ICP-AES	% of total	*in vivo*	% of soluble total	% of total
**Field soil site**	1-before	1.82 ±0.12	0.49 ±0.12	26.92	0.23 ±0.05	12.64	0.189 ±0.0046	10.38	0.029 ±0.011	60.42	1.59
	1-after	0.31 ±0.07	0.27 ±0.07	87.10	0.22 ±0.12	70.96	0.112 ±0.0011	36.13	0.096 ±0.029	85.71	30.96
	2-before	3.37 ±0.12	0.28 ±0.10	8.31	0.30 ±0.08	8.90	0.048 ±0.0015	1.42	0.034 ±0.003	70.83	1.01
	2-after	2.36 ±0.83	0.40 ±0.13	16.95	0.25 ±0.17	10.59	< LOD^†^	-	0.018 ±0.015	-	0.76
	3-before	1.80 ±0.07	0.45 ±0.21	25.00	0.24 ±0.04	13.33	0.049 ±0.0015	2.72	0.027 ±0.030	55.10	1.50
	3-after	1.62 ±0.16	0.22 ±0.09	13.58	0.18 ±0.07	11.11	< LOD^†^	-	0.025 ±0.019	-	1.54

* The values were represented as average with standard deviation as errors from more than 3 replicated tests, the unit of values was ppm (μg of cadmium/g of soil), % of total was the percentage of determined amount out of total cadmium in samples.

^†^ The concentration was lower than instrumental limit of detection.

As described above, the amount of bioavailable cadmium in amended LUFA soils quantified by both *in vivo* and *in vitro* assays was different, but it showed similar trend in relative amounts. In case of contaminated field soils, however, the trend of bioavailable cadmium determined in each assay was not matched each other (S3A and S3B Fig). The highest bioavailable cadmium was detected from the field soil site 1 before soil washing from *in vitro* assay, while field soil site 2 before soil washing was highest from *in vivo* assay. It would be reasoned that the physicochemical properties of site soils were different each other (S2 Table). For all that the quantification was performed based on same standard curve obtained by LUFA soil as a reference since it was practically impossible to have the proper reference for each field soil. Although it was unclear which data is correct at this moment, we believed the *in vitro* assay would be more accurate because it was not disrupted by matrix effects of soil particles.

To sum up, the amount of bioavailable cadmium was not decreased as much as total amount cadmium and the bioavailable portion was even increased in site 1 and 2 after soil-washing procedures. It might be explained by the harsh conditions of soil-washing procedures, sulfuric acid treatment in this case, disrupt the non-bioavailable cadmium tightly associated with soil particles to be bioavailable. Moreover, the heterogeneous nature and the physicochemical properties of soils would be expected to modulate the efficiency of the soil-washing process (S2 Table). Consequently, it was inferred that soil remediation procedure involving soil-washing should be selected under the consideration of the physicochemical properties of soils and the effectiveness should be evaluated in the aspect of bioavailability.

## Conclusions

In this study, we demonstrated the construction and characterization of WCBs harboring the promoter of zinc inducible operon fused with fluorescent proteins as a cadmium-specific bioreporter, and determined the bioavailability of cadmium in contaminated soils. The cadmium specificity of WCBs was investigated by structural analysis of the interaction between metal ions and regulatory proteins of the *znt*-operon, ZntR, using molecular simulation. The bioavailable cadmium in contaminated soils after soil-washing was determined using the WCB assay, along with novel strategies to minimize the matrix effects of soils. The amount of bioavailable cadmium was influenced by the physicochemical properties of soils, and a majority of bioavailable cadmium in contaminated soils was present in non-extractable states. The efficiency of total and bioavailable cadmium removal by soil-washing was investigated. The level of cadmium removal differed from site to site and was not proportional to the total amount of cadmium in soils because of soil heterogeneity. Therefore, the WCBs described here are valuable not only for sensitively detecting bioavailable cadmium and assessing the risk to living organisms but also to evaluate the effectiveness of remediation processes.

## Supporting Information

S1 Fig Structural analysis of the interaction between ZntR (PDB ID: 1Q08) and heavy metal ions. (A) 3-Dimensional structure of ZntR associated with 4 zinc ions after energy minimization using the Tripos force field. Arrows indicate zinc-binding sites of the ZntR dimer. (B) The interaction between zinc and residues in the metal binding site was analyzed by Ligplot. Zinc ions are indicated as green spheres in the center of residues.
(TIF)

S2 Fig The induction rates of eGFP and mCherry correspond to the growth of WCBs. (A) Growth curve of both WCB cells with (black dot) and without (white square) 5mg/L of Cd(II) as a function of time. The cell density was measured at 600 nm using a spectrophotometer. (B) Different expression rates of eGFP and mCherry as a function of time with 5 mg/L of Cd(II) induction. Cd(II) was added after 3 h cultivation, and the emission intensity of eGFP and mCherry were measured by fluorescence spectroscopy at 510 nm and 610 nm, respectively.
(TIF)

S3 Fig The induction coefficients of a WCB harboring pZnt-eGFP exposed to three sites of contaminated field soil samples before and after soil-washing. The induction coefficients for WCBs exposed to soils obtained by the *in vivo* assay (A) and *in vitro* assay (B). The induction coefficients for WCBs exposed to soil solutions (C).
(TIF)

S1 Table The distance (Å) between metal ions and atoms of residues in the metal binding site of ZntR measured by Sybyl 7.3 software. (DOCX)

S2 Table Physicochemical properties of soils tested in the present study (DOCX)
