# Supplementary figures and images for: Use of Tunable Whole-Cell Bioreporters to Assess Bioavailable Cadmium and Remediation Performance in Soils
Source: PLoS One. 2016 May 12;11(5):e0154506. doi: 10.1371/journal.pone.0154506 (PMC4865175; doi:10.1371/journal.pone.0154506)

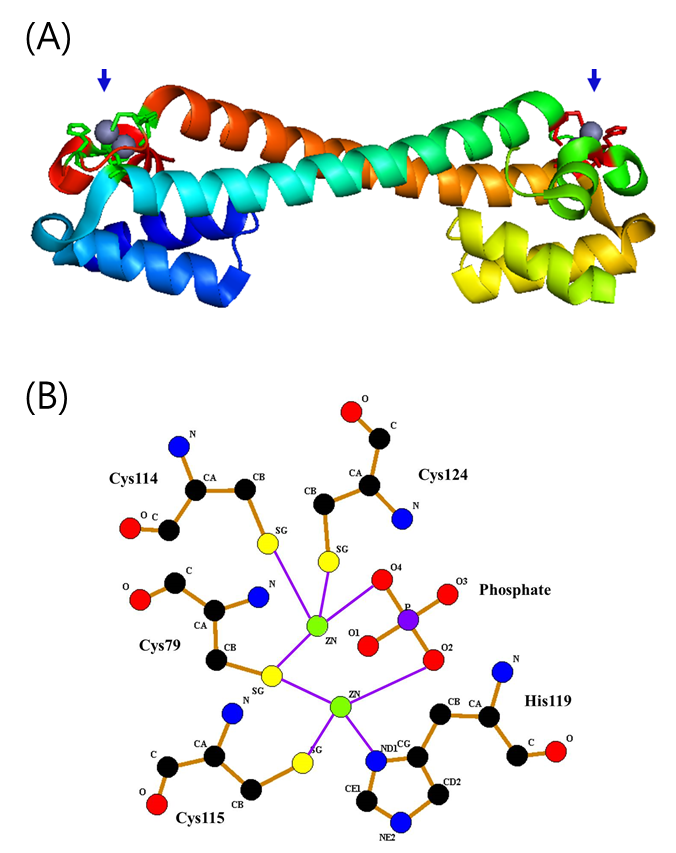

Supplement: S1 Fig — (A) 3-Dimensional structure of ZntR associated with 4 zinc ions after energy minimization using the Tripos force field. Arrows indicate zinc-binding sites of the ZntR dimer. (B) The interaction between zinc and residues in the metal binding site was analyzed by Ligplot. Zinc ions are indicated as green spheres in the center of residues. (TIF) [file pone.0154506.s001.tif]

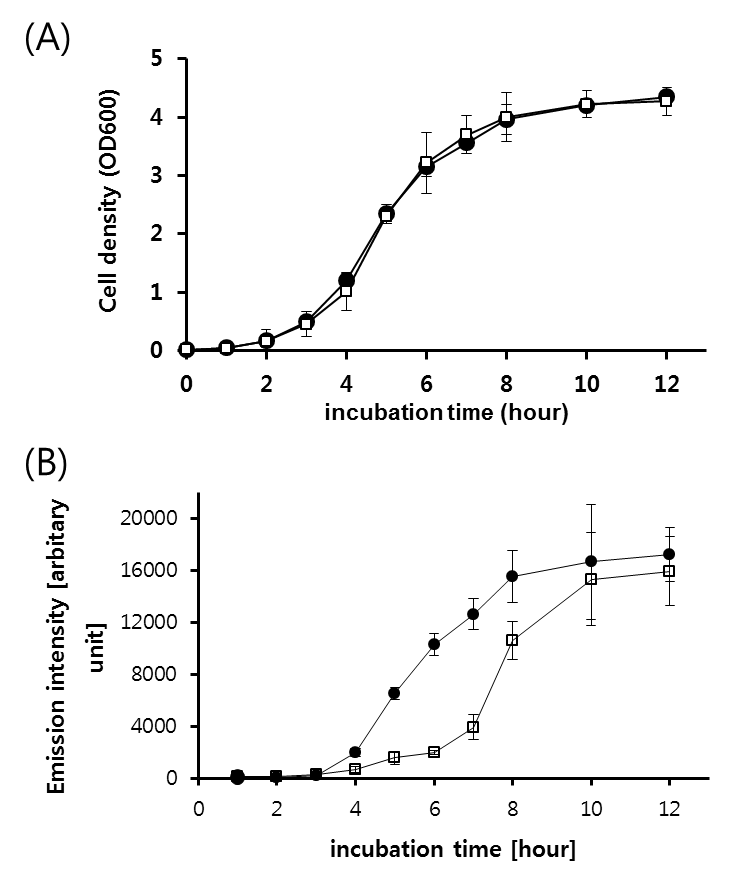

Supplement: S2 Fig — (A) Growth curve of both WCB cells with (black dot) and without (white square) 5mg/L of Cd(II) as a function of time. The cell density was measured at 600 nm using a spectrophotometer. (B) Different expression rates of eGFP and mCherry as a function of time with 5 mg/L of Cd(II) induction. Cd(II) was added after 3 h cultivation, and the emission intensity of eGFP and mCherry were measured by fluorescence spectroscopy at 510 nm and 610 nm, respectively. (TIF) [file pone.0154506.s002.tif]

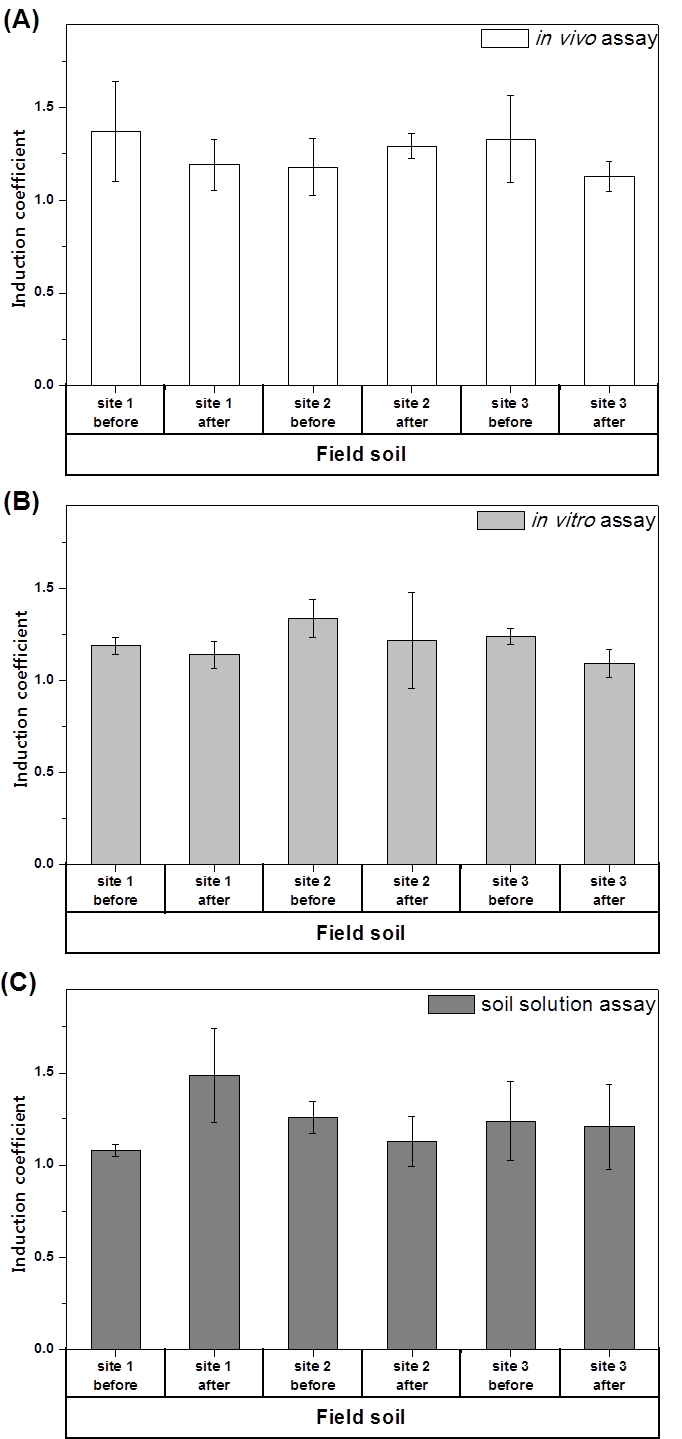

Supplement: S3 Fig — The induction coefficients for WCBs exposed to soils obtained by the in vivo assay (A) and in vitro assay (B). The induction coefficients for WCBs exposed to soil solutions (C). (TIF) [file pone.0154506.s003.tif]
