# Supplementary material for: Use of Tunable Whole-Cell Bioreporters to Assess Bioavailable Cadmium and Remediation Performance in Soils
Source: PLoS One. 2016 May 12;11(5):e0154506. doi: 10.1371/journal.pone.0154506 (PMC4865175; doi:10.1371/journal.pone.0154506)
Supplement: S1 Table — (DOCX) [file pone.0154506.s004.docx]

**S1 Table. The distance (Å) between metal ions and atoms of residues in the metal binding site of ZntR measured by Sybyl 7.3 software.**

|  | S_γ_ | | | | N_δ_ | O |
| --- | --- | --- | --- | --- | --- | --- |
|  | Cys79 | Cys114 | Cys115 | Cys124 | His119 | Phosphate |
| Zn1 | 2.08 | 2.09 | - | 2.08 | - | 1.87 |
| Zn2 | - | - | 2.09 | 2.08 | 1.97 | 1.86 |
| Cu1 | 2.08 | 2.09 | - | 2.08 | - | 1.87 |
| Cu2 | - | - | 2.09 | 2.08 | 1.97 | 1.86 |
| Cr1 | 2.21 | 2.22 | - | 2.21 | - | 1.99 |
| Cr2 | - | - | 2.21 | 2.21 | 2.08 | 1.99 |
| ^*^All atoms locate at A chain of homodimer of ZntR except Cys79 located at B chain.  ^**^The distance was measured after minimization processes with each heavy metal. | | | | | | |
