# Supplementary material for: Use of Tunable Whole-Cell Bioreporters to Assess Bioavailable Cadmium and Remediation Performance in Soils
Source: PLoS One. 2016 May 12;11(5):e0154506. doi: 10.1371/journal.pone.0154506 (PMC4865175; doi:10.1371/journal.pone.0154506)
Supplement: S2 Table — (DOCX) [file pone.0154506.s005.docx]

**S2 Table. Physicochemical properties of soils tested in the present study**

| **Indicators** | **Unit** | **LUFA** | **A-before** | **B-before** | **C-before** | **A**  **-after** | **B-**  **after** | **C-**  **after** |
| --- | --- | --- | --- | --- | --- | --- | --- | --- |
| Texture | - | Sand | Sand | Loamy sand | Sand | Loamy sand | Loamy sand | Loamy sand |
| Clay | % | 0.158 | 0.047 | 0.096 | 0.118 | 0.15 | 0.136 | 0.015 |
| Silt | % | 6.8 | 7.24 | 19.47 | 2.75 | 10.27 | 25.65 | 20.11 |
| Sand | % | 93.04 | 92.72 | 80.43 | 97.13 | 89.58 | 74.22 | 79.88 |
| Water contents | % | 25 | 18.4 | 29.78 | 15.57 | 20.6 | 46.11 | 21.24 |
| Aggregate stability | % | 73 | 74 | 59 | 88 | 69 | 58 | 63 |
| Bulk density | g/cm^3^ | 1.29 | 1.3 | 1.04 | 1.36 | 1.46 | 1.1 | 1.46 |
| Cation exchange capacity | meq/ 100g | 9.32 | 3.99 | 21.41 | 6.36 | 7.78 | 11.44 | 2.19 |
| Organic matter | % | 3.39 | 1.57 | 4.34 | 1.15 | 1.45 | 2.24 | 1.26 |
| pH | - | 6.71 | 7.35 | 6.3 | 8.29 | 4.17 | 5.87 | 8.96 |
| Total nitrogen | mg/kg | 2571 | 936 | 1107 | 767 | 465 | 945 | 107 |
| Total phosphorus | mg/kg | 230 | 251 | 233 | 272 | 193 | 99 | 173 |
| Water holding capacity | ml/g | 0.336 | 0.372 | 0.632 | 0.306 | 0.333 | 0.558 | 0.217 |
